# Exploring the Underlying Genetics of Craniofacial Morphology through Various Sources of Knowledge

**DOI:** 10.1155/2016/3054578

**Published:** 2016-12-08

**Authors:** Jasmien Roosenboom, Greet Hens, Brooke C. Mattern, Mark D. Shriver, Peter Claes

**Affiliations:** ^1^Department of Neurosciences, Experimental Otorhinolaryngology, KU Leuven, Herestraat 49, P.O. Box 721, 3000 Leuven, Belgium; ^2^Multidisciplinary Cleft Lip and Palate Team, UZ Leuven, Kapucijnenvoer 33, 3000 Leuven, Belgium; ^3^Department of Otorhinolaryngology, Head and Neck Surgery, Kapucijnenvoer 33, 3000 Leuven, Belgium; ^4^Department of Anthropology, Penn State University, 409 Carpenter Building, University Park, PA 16802, USA; ^5^Medical Image Computing, ESAT/PSI, Department of Electrical Engineering, KU Leuven, Medical Imaging Research Center, Herestraat 49, P.O. Box 7003, 3000 Leuven, Belgium

## Abstract

The craniofacial complex is the billboard of sorts containing information about sex, health, ancestry, kinship, genes, and environment. A thorough knowledge of the genes underlying craniofacial morphology is fundamental to understanding craniofacial biology and evolution. These genes can also provide an important foundation for practical efforts like predicting faces from DNA and phenotype-based facial diagnostics. In this work, we focus on the various sources of knowledge regarding the genes that affect patterns of craniofacial development. Although tremendous successes recently have been made using these sources in both methodology and biology, many challenges remain. Primary among these are precise phenotyping techniques and efficient modeling methods.

## 1. Introduction

The human face is unique among externally visible characteristics, largely due to the wealth of information on display for others to observe. Understanding the origins of human craniofacial variation within and across populations and between the sexes requires a better understanding of which genes and alleles are affecting craniofacial variation. While there is substantial evidence that human craniofacial variation is genetically determined, such as population and sex differences, family resemblances, and identical twins, the actual genetic architecture of craniofacial variation is poorly understood [1]. A better understanding of the genetic architecture of the human craniofacial complex could lead to a number of interesting scientific advances and applications. For example, although many genetic conditions that involve clinically significant patterns of facial development have been mapped, many individual cases remain undiagnosed genetically. A more thorough knowledge of the genetics of typical-range craniofacial development can and should help in the delineation of which genes underlie these conditions. Generally, this process has been inversed, with information on the genetic determinants of disorders serving to help identify candidate genes for investigations into typical-range craniofacial variation [2].

**Figure 1:**
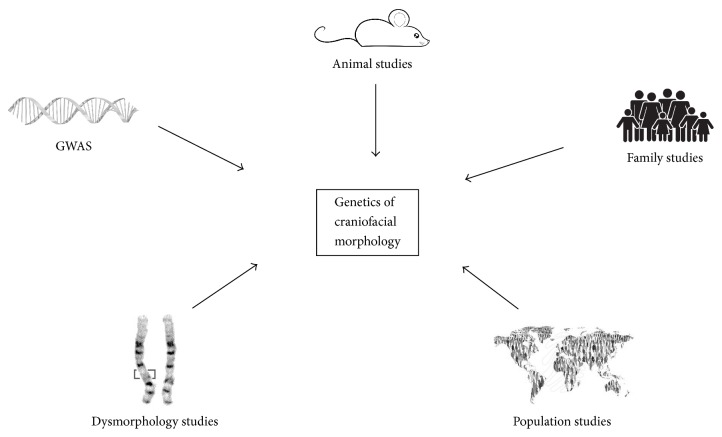
Various sources of knowledge to investigate the genetic etiology of craniofacial variation.

In recent years, questions regarding the genetic and environmental factors affecting variation in human craniofacial morphology have received increasing attention. Medical and clinical genetic research using family studies have proven foundational in establishing our understanding of which genes affect craniofacial variation. Testing these clinically relevant genes for significant effects in determining typical-range variation is one fruitful avenue of investigation, as are twin studies and nonhuman animal studies [3]. As the result of breakthroughs in genotyping technologies, large publicly funded projects (like the Human Genome Project [4], Hapmap [5], and 1000 Genomes [6]), and methodological advances in statistical genetics, many genome-wide association studies (GWAS, looking at a genome-wide set of variants in a population to see if a variant is associated with a trait) have been completed in the past few years. This review provides an overview of the various sources of knowledge that can yield information regarding the genes and alleles that affect patterns of craniofacial development (Figure 1). Differences among alternate approaches, in terms of their relevance, challenges, and limitations, are discussed. Finally, avenues towards the future are outlined.

## 2. Animal Studies

As with many human traits and diseases, animal models are very useful in understanding the genetic basis of variation in the human craniofacial complex. Research using model organisms such as mice, dogs, and zebrafish has provided insights into several processes involved in craniofacial development. The embryonic faces of the various amniote species all show a high degree of similarity, attesting to both the developmental conservation and rationale of comparative studies [7]. Embryological studies in both mouse and chicken have shown a major role of sonic hedgehog (*shh*) and bone morphogenic protein (*bmp*) signaling in the development of the frontonasal zone [7]. Genes in these pathways are shown to affect orofacial clefting in humans [8], which is also well described in animal models [9–11]. Research on a canine model of Pierre Robin Sequence revealed that a LINE-1 insertion in the homologue to the human* DLX6* gene is responsible for cleft palate and associated mandibular abnormalities [12]. Sequencing of* DLX5* and* DLX6* in a cohort of humans with isolated cleft palate has shown causal effects of missense mutations in* DLX5*. A knockout of* Nol11* in Xenopus Tropicalis leads to an increased apoptosis of cranial neural crest cells and thus to craniofacial abnormalities [13]. This effect could be rescued to some extent by a knockdown of* p53*. In humans, this defect could be linked to craniofacial abnormalities seen in Treacher Collins syndrome (OMIM #154500). These findings provide examples of the versatility of animal studies and their ongoing relevance to understanding human craniofacial variation. Moreover, model organisms enable gene knock-out and knock-in experiments which can provide more in-depth information on the functional effects of genetic variants. For example, Attanasio et al. performed some large-scale transgenic analysis to identify over 4000 long-distance enhancers that significantly affect normal craniofacial development in mice [14]. A Cre/loxP conditional knock-out approach in mice showed that* foxf2* is required for normal palatogenesis in mice [15].

Not only can the use of laboratory animals be helpful in investigating the genetic etiology of craniofacial variation, but also studies of domesticated and wild animals can give new insights [16]. Lamichhaney et al., for example, have looked into the evolution of Darwin's finches and their beaks [17]. A genome-wide scan comparing different species of finches showed that variation in beak morphology can be due to variation in* ALX1*. In humans, loss of* ALX1* is causing frontonasal dysplasia (OMIM #136760) [18]. In a recently published study, Pallares and colleagues were able to map within population variance of craniofacial shape of inbred mice [19]. Furthermore, they identified 17 loci responsible for variation in skull shape and eight loci responsible for variation in mandible shape of these mice, with* Mn1* as a key gene in skull formation and within population shape variation.

## 3. Dysmorphology Studies

At the moment, 8,201 phenotypes are described in the OMIM database (Online Mendelian Inheritance in Man, http://www.omim.org/statistics/entry), of which 4,787 have a known molecular basis. Approximately 32% of the inherited human disorders are associated with atypical craniofacial characteristics [20–22]. These “face signatures” (i.e., the face shape difference normalized against age and sex matched controls) can provide the additional clues for clinical diagnoses of genetic syndromes [23, 24].

When trying to identify the genetic cause of these syndromes, genetic data of affected and unaffected family members can be compared. Variants that occur in affected family members, but not in unaffected family members, can be causal for the syndrome or can be in linkage disequilibrium (the nonrandom association of alleles of different loci) with causal genetic variants for the condition. Linkage analysis is a means by which the coinheritance of makers and diagnostic status (affected versus unaffected) are formally modeled and tested for statistical significance. Linkage analysis for craniofacial conditions has been tremendously successful and has provided much of what we currently know about the genes affecting human craniofacial morphology.

Although linkage analysis studies have proven to be very useful in defining the genes underlying atypical patterns of craniofacial development, researches on how exactly (in 3D) the faces of affected and unaffected persons differ have lagged behind mapping studies. A small group of researchers have been investigating a subset of conditions involving the development of the face using 3D morphometrics (quantitative analysis of form) and have illustrated the difference between the unaffected and affected faces as face signatures. Face signatures have proven important in understanding the effects on the face of some primarily psychiatric or neurological disorders such as epilepsy [25]. Developmentally, the face evolves in concert with the brain, with each influencing the development of the other and sharing genetic signaling pathways [26]: in developmental neurological disorders the phrase “the face predicts the brain” is commonly used [27]. Since the genetic causes of many syndromes are partially understood, investigating the faces of these syndromic patients can be very informative. The genes and gene regions involved in patterns of atypical craniofacial development may also be involved in typical-range craniofacial variation.

De novo generation of a syndrome with a nonspecific craniofacial morphology can provide insights into the genetic etiology of craniofacial variation, by finding the location of the de novo mutation [20, 28]. The gene region where this mutation is located can either be functionally responsible for the craniofacial trait or in linkage disequilibrium with the variant that is directly affecting the phenotype. Another opportunity involves investigating whether or not genes and alleles that affect atypical patterns of craniofacial development also affect typical-range facial variation. An example from the literature is the* PAX3* gene which can significantly affect typical-range facial variation including the breadth of the nasal bridge [2, 29]. Clinically significant mutations in* PAX3 *can lead to Waardenburg syndrome (OMIM #193500) which features hypertelorism and broad nasal ridges. These facial features were also noticed by Claes et al. in their attempt to replicate SNPs rs7559271 and rs974448 in* PAX3*, using spatially dense geometric analysis techniques on normal-range faces [2].

In addition to syndromes, some other patterns of atypical craniofacial development also have been widely investigated. In fact, syndromic (associated with other anomalies in the context of a known syndrome) or not, about 3% of newborns have a “major physical anomaly,” meaning a physical anomaly that has cosmetic or functional significance [30]. The best-known example is nonsyndromic cleft lip with or without cleft palate (CL/P) with an incidence of 1/700. This congenital condition has a multifactorial etiology, with both environmental and genetic risk factors [31]. It has widely been investigated and studies have revealed several genes affecting craniofacial morphology [7, 32].

Further, conditions such as craniosynostosis can give insight in the genetic etiology of skull development. Craniosynostosis occurs through premature closure of the skull sutures and can be recognized by an abnormal skull shape in the newborn. Genes responsible for this condition are likely to play a role in typical skull formation [33–35].

Dysmorphology studies have been and will remain valuable for the identification of individual genes or groups of genes affecting craniofacial morphology. A complete overview of these studies is not within the scope of this work and is often specifically described per condition [36–38]. The main challenge lies in the limited availability of persons expressing the same or similar craniofacial configurations. Larger more collaborative efforts for collecting data of these patients may be necessary. If 3D images of sufficient numbers of patients with known genetic background can be compared to unaffected persons, the facial changes diagnostic of the craniofacial conditions can be distinguished from typical-range effects.

## 4. Population Studies

Although craniofacial variation both within and among populations is clearly evident, systematic analyses of patterns of this variation using modern morphometric methods have been limited. Phenotypic variation, like genetic variation, is the result of four evolutionary forces, namely, genetic drift, natural selection (both ecological and sexual selection), admixture (interbreeding between two previously isolated populations), and mutation [39]. Evolutionary studies on the morphology of the human skull exemplify some of the approaches to understanding the evolution of complex traits [40]. Skeletal analyses suggest that the primary evolutionary factor leading to population differentiation and indeed most genetic variation in contemporary human populations has been genetic drift: one study showed that 90% of the variation in 3D craniofacial landmark coordinates is shared across populations while only 10% of the total variation is between population variation [41]. Claes and colleagues showed that 9.6% of the total facial variation in an African/European mixed population was due to variation in genetic ancestry. As such, between population studies, like admixture mapping, will likely provide only a subset of all of the genes affecting craniofacial variation.

In contrast to the skeletal component, the soft tissue component of the face may have been affected more by nonneutral evolutionary processes [39] such as sexual selection and local adaptation due to its direct exposure to the environment. For example, one study presented results suggesting that variations in the nose and brow area across four Eurasian populations (Han Chinese, Tibetans, Uyghur, and Europeans) were higher than expected under genetic drift alone [39]. These authors propose that the European nose may be an adaptation to colder climates. Furthermore, they speculated that the enlarged brow area in Europeans might have been influenced by adaptation to specific diets. Sheehan and Nachman showed that also selection for individual identity signals has shaped patterns of human facial diversity [42]. Another recent study investigated the patterns of sexual dimorphism in a sample of the faces of persons of European-derived ancestry and showed large sex differences in several parts of the face including the chin, brow ridge, and upper cheek region [43]. Comparing these patterns with those reported in Claes et al., 2012 [43], it seems that there are differences in the patterns of sexual dimorphism between a primarily European sample and the approximately half West African/half European sample. Differences are seen in the brow ridges, noses, and the chins.

Once the functional genes and alleles have been identified, molecular evolutionary genetic approaches to investigate the timing and geographical locations where gene frequency changes occurred can help us understand the mechanisms behind changes in the human craniofacial complex across evolutionary time.

## 5. Familial Studies

Familial resemblance in facial features is one of the main indications that craniofacial shape is genetically regulated. Twin studies are well-known and have been used extensively to study the heritability of a wide range of traits and behaviors [44]. Monozygotic twins share nearly identical DNA sequences and are the extreme example of familial resemblance providing striking examples of facial similarity. When monozygotic twins are studied in contrast to dizygotic twins, the extent to which genetic factors affect craniofacial morphology can be investigated. An added advantage of twins is that they are at the same age, such that comparisons between facial images of twins are not confounded by growth or aging [45–47]. Recently, a large twin study investigated which parts of the face were prone to genetic and environmental influences [48]. This showed that genetic factors mostly determine facial size, nasal shape, lips prominence, and interocular distance, while mandibular ramus height and horizontal facial asymmetry are influenced by environmental factors.

Twin and other family studies of craniofacial shape show a moderate to high degree of heritability for a substantial set of craniofacial traits [46, 49–51]. Facial height, width, and nasal features, in particular, are more genetically determined than is facial depth [51]. Furthermore, local facial features with high heritability include the orbits, nose, jaw, and teeth. These studies differ in several respects, namely, the study design (twins or parent-offspring), the data acquired (radiographs, 3D facial surface scans, medical MRI, or CT), the sample sizes, landmark density, and the type of measurements extracted and analyzed (e.g., interlandmark distances or principal components). Furthermore, from these studies it is clear that some facial regions are more strongly influenced by the environment than by genes. One example is the variability typically noted in the cheeks due to body weight change and/or aging [52].

## 6. Genome-Wide Association Studies (GWAS)

Linkage analysis in families is one way to identify the genetic loci underlying a trait of interest. Genetic association is the other major means for identifying the alleles affecting variation in a trait and is usually employed in a GWAS analytical framework. In a GWAS, phenotype-genotype associations are investigated in large population samples [53]. Both qualitative traits (affected versus unaffected) and continuously distributed traits like height can be investigated using GWAS. Although very well established in research to the genetic origin of disease, GWAS as an attempt to discover genetic variants responsible for craniofacial morphology is still in its infancy.

Liu et al. and Paternoster et al. published the first two GWAS on typical-range craniofacial genetics in 2012 [29, 54]. The GWAS by Liu et al. identified five loci that are associated with variation in facial morphology in Europeans [54]. They suggested five candidate genes:* PRDM16*,* TP63*,* C5orf5N*,* COL17A1*, and* PAX3*. The* PAX3* gene was also identified in the GWAS reported by Paternoster et al. and was the first typical-range facial gene to be confirmed across independent studies [29]. Both GWAS were carried out in Europeans. Recently, three additional GWAS have been published. Adhikari et al. could associate four different genomic regions with three nose-related traits and with chin retrusion analyzing 2D frontal photographs of about 6000 subjects of Latin-American descent [55]. The strongest associations in the genomic regions were observed in the* EDAR, DCHS2, RUNX2*, and* GLI3* genes. Furthermore, they were able to replicate the previously described association of nasion position and* PAX3*. Shaffer et al. conducted a GWAS analyzing 20 quantitative facial measurements on 3D images of a cohort of about 3100 American individuals of European descent. They found six regions associated with variable facial traits, in which several genes are located which are known to be associated with craniofacial development:* MAFB, PAX9, MIPOL1, ALX3, HDAC8, *and* PAX1 *[56]. Furthermore, they were able to replicate the correlation between nasal ala length and SNPs in* CACNA2D3 *and* PRDM16* and between intercanthal width and SNP rs7559271 in* PAX3*. A third recently published GWAS looked at 3D images of African children and found an association of* SCHIP1 *and* PDE8A* with facial shape and size [57]. They were also able to show clear expression of these genes in the developing mouse face, indicating a role for these genes in normal craniofacial development. SNPs that were identified in previous described GWAS are listed in Table 1.

Disease GWAS findings also contribute additional insights on the genetics of the face. For instance, Leslie et al. reported on a GWAS in 1,409 CL/P trios, identifying functional variants for CL/P in or near three genes:* PAX7*,* FGFR2*, and* NOG* [58]. They confirmed the importance of these variants using targeted sequencing and functional analyses using in vitro and in vivo assays. Every significant gene or allele finding can and should be investigated through further association studies and linkage studies, population genetic studies, functional genomic studies, and animal model experiments. For instance, to further examine GWAS findings of a role of* PAX7*, its function in mice neural crest was recently investigated indicating that* PAX7* plays a role in craniofacial development in mammals, especially by its presence in the neural crest lineage [59].

Although GWAS studies are useful means for identifying gene variants affecting both traits and disease risks, an important limitation in association testing is that the statistical power to detect an effect decreases with the decreasing allele frequency [53]. Thus, only relatively common alleles (i.e., those showing frequencies > 5% to 10%) can be tested and large databases are needed. Furthermore, significant efforts are required to collect sufficient (preferably 3D) images of participants of the same population background, which are then processed in a similar manner, for instance, using automated landmarking instead of manual landmarking. Additional challenges include the multiple testing problem as well as observer biased and limited descriptions of the facial phenotype in current GWAS. For example, in the recently reported GWAS on typical-range facial variation [29, 54], the facial phenotype was summarized as a set of univariate variables. Importantly, craniofacial variation among individuals can rarely be described with either qualitative or single quantitative variables. This problem of a highly multivariate/multipartite trait may be addressed using multivariate GWAS methods [60], consensus-face based comparisons [61], and partial least squares regression (PSLR) based methods like bootstrapped response-based imputation modeling (BRIM) [62]. In the future, it might be valuable to include different population backgrounds in very large GWAS in order to increase our knowledge about population-specific genetic influences on craniofacial morphology. Admixed populations offer a distinct advantage in these efforts as they include the gene pools of two populations in a single group that should share more elements of environmental exposure than the populations separately.

## 7. Phenotyping

Advancements in the discovery of genes affecting craniofacial variation rely in part on the ability to accurately capture and define the morphological complexity of the human craniofacial complex. Data on morphology phenotypes can be thoroughly collected through imaging. For the human face, both 3D surface scanners and medical imaging scanners provide excellent technological means to capture shape and appearance information. Three-dimensional facial surface scanning, such as laser surface and photogrammetric imaging, is well-suited to capturing facial form. This is especially important in healthy subjects because these scanning techniques are noninvasive in contrast to medical computer tomography and X-ray imaging, which use ionizing radiation. However, the major advantage of the latter systems is that they can be used to capture the bony structures of the craniofacial complex. Although each phenotyping approach has its advantages, comparison of different kinds of images and measurements is challenging [2].

Besides the advancement of acquisition techniques to capture facial morphology, the analytic methods to describe craniofacial shape must also gain in resolution, precision, and power. Shape is unfortunately often still described using only a sparse set of specific biological landmarks (these being defined as “a point of correspondence on an object that matches between and within populations”), which are indicated manually on each image. This step can introduce operator error in the placement of landmarks, which can lead to contrasting study outcomes [63]. Furthermore, manual indication is time-consuming and requires skill and training. Finally, because some anatomic regions of the face lack discrete features, only a limited number of landmarks can be used, as a consequence salient features of the facial shape are overlooked [64, 65]. Measurements from sparse landmarks such as distances, angles, and ratios, also known as conventional morphometric analysis [66], had been the primary approach to investigating craniofacial features. However, this conventional morphometric approach oversimplifies the 3D craniofacial complex such that facial characteristics of interest may be discounted [67]. The previously described GWAS on typical-range facial variation [29, 54–57] and all of the familial studies on facial heritability thus far started from sparse landmark representation of facial shape. Subsequently, interlandmark distances and/or angles are extracted in combination with a set of principal coordinates using principal component analysis on the landmark coordinates. However it is unfounded to assume that each PC represents a distinct and plausible morphological facial trait. Furthermore, all measurements, distances, and/or principal coordinates are selected* a priori*, meaning that many measures will need to be looked at in order to describe the effects of even one independent variable.

Several extensions to spatially dense landmarks have been proposed, including quasilandmarks and semilandmarks [63]. The main advantage of spatially denser set of landmarks is that they provide more coverage and therefore a fuller description of shapes [68]. The challenge however is that the number of shape variables almost always exceeds the number of observations leading to theoretical limitations on the use of some statistical methods [69]. Shape regression, for example, is a useful technique to investigate the effect of an independent variable of interest (in this case genetic, familial, and/or population information) on facial morphology. When working with spatially dense shape representations in contrast to ordinary least-square regression more advanced techniques such as partial least squares regression should be used to gain additional facial information. Additional techniques, such as BRIM [62], further aid to increase statistical power to detect genuine genotype-phenotype correlations.

In the interpretation of the phenotype, one has to be cautious and attentive. The term “phenotype” in a clinical setting is often used to indicate characteristics that are deviating from the “normal” or typical morphology, physiology, and behavior [24]. This gives rise to questions of what is typical. Sometimes, phenotypic features are associated with a genetic disease, without being a deviation of the standard. Endophenotypes, for example, are characteristics (behavioral or anatomical) that are associated with a condition and are present in nonaffected family members. They are considered to be an expression of underlying susceptibility genes for the condition. For instance, in orofacial clefting, endophenotypic facial features, such as hypertelorism and midface retrusion, are described in nonaffected first-degree relatives of patients [70, 71]. Objective characterization of these endophenotypes and their underlying genetics can therefore indicate new candidate genes for this type of multifactorial conditions.

## 8. Discussion

The interpretation of the genotype is regulated on many different levels, where in addition to genetic variation, epigenetic and distance regulators play a major role as well [14]. The epigenetically switching on and off of genes and gene activity can lead to significant changes on the phenotype level. Therefore, it is important to combine the information retrieved from GWAS with underlying molecular actions on a genetic and an epigenetic level [72]. Only then, a correct interpretation and verification of the GWAS results are possible. Moreover, gene dosage also has an effect on craniofacial development, which is important in syndromes with a causal copy number variation and also in typical craniofacial development [73]. Another challenge is that an opposite copy number effect (deletion versus duplication for instance) does not necessarily cause an opposite facial effect, although recently Hammond et al. reported opposite effects on facial morphology in opposite copy number variation of genes [73]. In conclusion, unfolding genotype-phenotype correlations on craniofacial morphology is clearly challenging due to its genetic complexity [74].

While progress has been made in the uncovering of genes affecting craniofacial variation using a traditional “predefined trait” approach, the adaptation of a phenomic point of view, beyond “phenotyping as usual” [75], has much to offer these efforts. Large-scale research, multidisciplinary, and up-to-date methods and analysis techniques are essential to summarize phenotypes as complex as human craniofacial morphology. Therefore, collaboration not only between but also within institutes (e.g. between medical doctors, fundamental scientists, engineers, statisticians, and informaticians) is indispensable [76].

When comparing the findings from different types of studies, it is important to keep in mind that there can be many differences among studies investigating the same hypothesis. Not only study design (e.g., parent-offspring studies and animal studies) but also the method of data acquisition (e.g., radiographs and anthropometric measurements), differences in sample size, age of the cohort, and the method of analysis can influence results obtained. Therefore, it is not always appropriate to simply compare the findings and conclusions across several studies. An introduction of guidelines for more standardized research methods can be useful as well as a central database to register results of research on typical-range and clinically significant craniofacial genetics. A good example of such a database is FaceBase (https://www.facebase.org/) [77]. It is also useful to register negative research results, to avoid unnecessary replication of such studies that often involve expensive and time-consuming data sampling efforts. It would even be more optimal to have a common set of participants on which all current and new methods can be compared and validated.

Through existing and future investigations of facial heritability, craniofacial evolution, and individual gene effects, a better understanding of the genetic architecture of craniofacial morphology is certainly anticipated. With increased resolution, precision, and power of existing and future analysis techniques we may well get to the point in which our understanding of the genetic determinants of craniofacial variation can lead to practical facial predictions from DNA. First attempts in this direction were recently demonstrated by Claes et al. and by Fagertun et al. [62, 78]. Although novel and promising, the work is highly preliminary and future refinement of the prediction technique is essential.

In conclusion, the genetic architecture of craniofacial morphology is complex and a challenge to unravel. We expect that many genes, with alleles causing small average effects, and both gene-gene and gene-environment interactions will play important roles in determining craniofacial variation. Future studies that investigate different aspects of craniofacial variation in the context of genetic variation in several human populations and animal models are indispensable. Given the recent development of new methods for more fully capturing and modeling craniofacial variation, we can expect new genes to result from both typical and atypical trait-based research. Furthermore, although analytic techniques for the genome but more importantly for the facial phenome are certainly advancing, future computational developments in bioinformatics, computational imaging, and developmental biology, for example, are still required. Nevertheless, the methodological advances and initial results provide both the impetus and analytical framework for future multidisciplinary studies to investigate the genetic determinants of human facial variation [79].

## Figures and Tables

**Table 1:** SNPs proven to have an influence on facial morphology, as shown by different published GWAS.

Publication	SNP	Gene	Effect
Paternoster et al., 2012	rs7559271	*PAX3*	Nasion–midendocanthion distance

Liu et al., 2012	rs4648379	*PRDM16*	Nose width and nose height
	rs168686344, rs12694574, rs974448	*PAX3*	Distance between eyeballs and nasion
	rs17447439	*TP63*	Distance between the eyeballs
	rs6555969	*C5orf50*	Nasion position
	rs805722	*COL17A1*	Distance between eyeballs and nasion

Adhikari et al., 2016	rs12644248	*DCHS2*	Columella inclination
	rs1852985	*RUNX2*	Nose bridge breadth
	rs17660804	*GLI3*	Nose wing breadth
	rs927833	*PAX1*	Nose wing breadth
	rs3827760	*EDAR*	Chin protrusion

Shaffer et al., 2016	rs6129564	*MAFB*	Cranial base width
	rs17106852	*PAX9*	Cranial base width
	rs17106852	*MIPOL1*	Cranial base width
	rs619686	*ALX3*	Intercanthal width
	rs11093404	*HDAC8*	Intercanthal width
	rs2424399	*PAX1*	Nasal width

Cole et al., 2016	rs79909949	*SCHIP1*	Centroid size
	rs12909111, rs12908400	*PDE8A*	Allometry
